# microRNA-200c/141 upregulates SerpinB2 to promote breast cancer cell metastasis and reduce patient survival

**DOI:** 10.18632/oncotarget.15680

**Published:** 2017-02-24

**Authors:** Tiefeng Jin, Hoe Suk Kim, Sul Ki Choi, Eun Hye Hwang, Jisu Woo, Han Suk Ryu, Kwangsoo Kim, Aree Moon, Woo Kyung Moon

**Affiliations:** ^1^ Department of Radiology, Seoul National University Hospital, Jongno-gu, Seoul 03080, Korea; ^2^ Department of Pathology & Cancer Research Center, Yanbian University Medical College, Yanji 133002, China; ^3^ Department of Biomedical Science, Seoul National University College of Medicine, Seoul National University, Jongno-gu, Seoul 03080, Korea; ^4^ Department of Pathology, Seoul National University Hospital, Jongno-gu, Seoul 03080, Korea; ^5^ Division of Clinical Bioinformatics, Biomedical Research Institute, Seoul National University Hospital, Jongno-gu, Seoul 03080, Korea; ^6^ Duksung Innovative Drug Center College of Pharmacy, Duksung Women's University, Dobong-gu, Seoul 01369, Korea

**Keywords:** SerpinB2, SerpinE1, metastasis, microRNA-200c/141, triple negative breast cancer

## Abstract

The microRNA-200 (miR-200) family is associated with tumor metastasis and poor patient prognosis. We found that miR-200c/141 cluster overexpression upregulated SerpinB2 in the MDA-MB-231 triple-negative (TN) breast cancer cell line. We observed transcription factor (c-Jun, c-Fos, and FosB) upregulation, nuclear localization of c-Jun, and increased SerpinB2 promoter-directed chloramphenicol acetyltransferase activity in miR-200c/141 cluster-overexpressing cells relative to controls. Additionally, miR-124a and miR-26b, which directly target SepinB2, were downregulated compared to controls. In mouse xenograft models, miR-200c/141 cluster overexpression promoted lymph node and lung metastasis, and siRNA-mediated SerpinB2 knockdown decreased lung metastasis, suggesting that SerpinB2 mediates miR-200c/141-induced lung metastasis. We also explored the clinical significance of SerpinB2 protein status through analysis of primary breast tumor samples and The Cancer Genome Atlas (TCGA) data. High SerpinB2 levels were associated with reduced survival and increased lymph node metastasis in breast cancer patients. SerpinB2 was overexpressed in the TN breast cancer subtype as compared to the luminal subtype. The present study demonstrates that SerpinB2 promotes miR-200c/141 cluster overexpression-induced breast cancer cell metastasis, and SerpinB2 overexpression correlates with increased metastatic potential and unfavorable outcomes in breast cancer patients. SerpinB2 may be a useful biomarker for assessing metastasis risk in breast cancer patients.

## INTRODUCTION

The microRNA-200 (miR-200) family consists of five members in two clusters, miR-200c/141 and miR-200b/a/429, which likely target different, but sometimes overlapping genes, thereby regulating many biological processes as oncomiRs or tumor suppressors [1, 2]. Individual miR-200 family member target genes appear to be cancer type- and context-dependent [3]. In many cancers, miR-200 member expression status serves as a surrogate marker for metastasis, response to drug treatments, and patient outcomes [4–9]. miR-200c overexpression predicts poor outcome in patients with hormone receptor-negative breast cancer [9]. miR-141 overexpression is observed in metastatic cases and is associated with poor outcome in breast cancer (BC) patients [10, 11]. Despite advances in our understanding of BC-related miR-200 members, their precise roles in breast cancer cell (BCC) metastasis are largely unknown, in part because each miR-200 has several putative targets with disparate functions. The underlying mechanisms by which miR-200 overexpression promotes metastasis require further study, and genes that serve as intermediaries in miR-200-associated metastasis are yet be identified.

Our preliminary studies showed that miR-200c/141 cluster overexpression in a triple-negative (TN) human BCC line, MDA-MB-231, which lacks estrogen receptor (ER), progesterone receptor (PR) and human epidermal receptor 2 (HER2) expression, enhances the migration and invasion abilities [12], upregulates SerpinB2 and promotes lymph node (LN) and lung metastasis in mouse models. SerpinB2, also known as plasminogen activator inhibitor-2 (PAI-2), inhibits urokinase plasminogen activator (uPA) activity [13–15]. SerpinB2 is overexpressed in tumor tissues relative to corresponding normal tissues, and has been clinically associated with poor prognosis in primary breast and other solid cancers [16–18]. SerpinB2 may promote tumorigenesis via inhibition of cell apoptosis [17, 19, 20], and reportedly promotes metastasis by fostering vascular co-option [21]. However, the role of SerpinB2 as a prognosis marker in tumor progression or suppression remains controversial.

The purpose of this study was to clarify whether SerpinB2 is involved in metastasis promoted by miR-200c/141 cluster overexpression in mouse xenograft models. Additionally, we investigated whether SerpinB2 expression is associated with patient clinicopathological features and outcomes using primary BC tissues and The Cancer Genome Atlas (TCGA) data.

## RESULTS

### miR-200c/141 cluster overexpression upregulates SerpinB2

**Figure 1:**
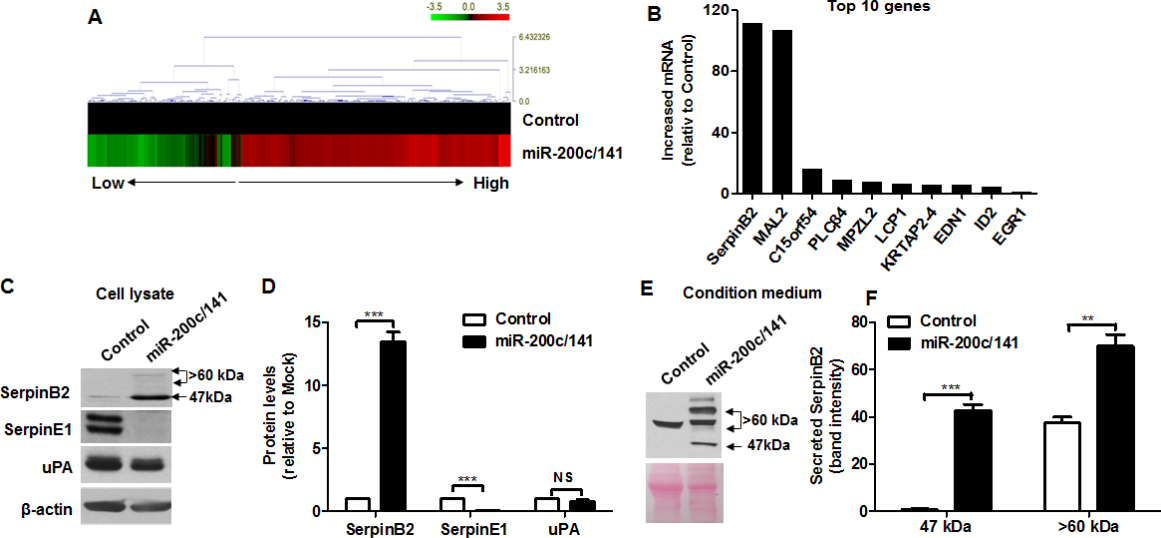
miR-200c/141 cluster overexpression upregulates SerpinB2 Heat map of relative gene expression in control and MDA-MB-231^miR-200c/141^ cells **(A)** Real-time RT-PCR analysis of the top 10 genes (SerpinB2, MAL2, C15orf54, PLCβ4, MPZL2, LCP1, KRTAP2-4, EDN1, ID2, and EGR1) **(B)** Representative images **(C)** and complete data **(D)** from SerpinB2, SerpinE1, and uPA western blots with control and MDA-MB-231^miR-200c/141^ cell lysates Representative SerpinB2 western blot with control and MDA-MB-231^miR-200c/141^ cell CM **(E)** Data from western blots of both SerpinB2 forms secreted from control and MDA-MB-231^miR-200c/141^ cells **(F)** ***P<0.001. NS, not significant.

Levels of miR-200c (201.88 ± 7.92-fold) and miR-141 (51.26 ± 3.48-fold) assessed by real-time RT-PCR were higher in MDA-MB-231 cells overexpressed miR-200c/141 (MDA-MB-231^miR-200c/141^ cells) than MDA-MB-231 cells (control) (*P*<0.0001) (Supplementary Figure 1). Up- or downregulated genes from the cDNA microarray experiments are represented as heat maps (Figure 1A). From real-time RT-PCR analyses verifying the cDNA microarray results, the top 10 genes upregulated by miR-200c/141 were as follows: SerpinB2 (114.52 ± 4.0-fold); MAL2 (101.56 ± 3.8-fold); C15orf54 (15.94 ± 0.3-fold); PLCβ4 (9.22 ± 0.27-fold); MPZL2 (7.55 ± 0.15-fold); LCP1 (6.33 ± 0.02-fold); KRTAP2-4 (6.12 ± 0.34-fold); EDN1 (5.41 ± 0.13-fold); ID2 (4.24 ± 0.13-fold); and EGR1 (1.14 ± 0.18-fold) (Figure 1B).

Figure 1C shows levels of SerpinB2 (47-kDa and >60-kDa forms) SerpinE1, and uPA in control and MDA-MB-231^miR-200c/141^ cell lysates. Both SerpinB2 forms were upregulated in MDA-MB-231^miR-200c/141^ cells (13.48 ± 0.74-fold, *P*<0.0001) relative to controls, whereas SerpinE1 was downregulated (0.056 ± 0.02-fold, *P*<0.0001) (Figure 1D). Slight uPA downregulation was observed in MDA-MB-231^miR-200c/141^ cells, but was not significant (0.79 ± 0.13-fold, *P*=0.19) (Figure 1D). A 47-kDa SerpinB2 was detected in MDA-MB-231^miR-200c/141^ cell conditioned medium (CM), but large forms (>60-kDa) were highly detected in both control and MDA-MB-231^miR-200c/141^ cell CM (Figure 1E). Both SerpinB2 forms were present at higher levels in MDA-MB-231^miR-200c/141^ cells relative to controls (*P*<0.0001) (Figure 1F).

We observed increases in transcription factor expression (c-Jun, c-Fos and FosB mRNAs), nuclear localization of the transcription factor, c-Jun, and SerpinB2 promoter-directed chloramphenicol acetyltransferase (CAT) activity in MDA-MB-231^miR-200c/141^ cells relative to controls (Supplementary Figures 2A, 2B and 2C). miR-124a and miR-26b, which directly target SepinB2, were downregulated in MDA-MB-231^miR-200c/141^ cells compared to controls (Supplementary Figure 2D). Together, these results showed that miR-200c/141 overexpression increases SerpinB2 indirectly by regulating SerpinB2 transcription factors and miRNAs in MDA-MB-231 cells. Although a significantly higher level of miR-200c was observed in HCC-38^miR-200c/141^, Hs578T^miR-200c/141^, and MCF-7^miR-200c/141^ cells relative to controls, SerpinB2, c-Jun and c-Fos mRNAs were downregulated (Supplementary Figures 3A, 3B, and 3C). miR-124a was upregulated in Hs578T^miR-200c/141^ and in HCC-38^miR-200c/141^ cells but was downregulated in MCF-7^miR-200c/141^ cells compared to controls (Supplementary Figure 3C). miR-26b was upregulated in only Hs578T^miR-200c/141^ cells compared to control (Supplementary Figure 3C).

### miR-200c/141 cluster overexpression promotes BCC lung and LN metastasis in mice

**Figure 2:**
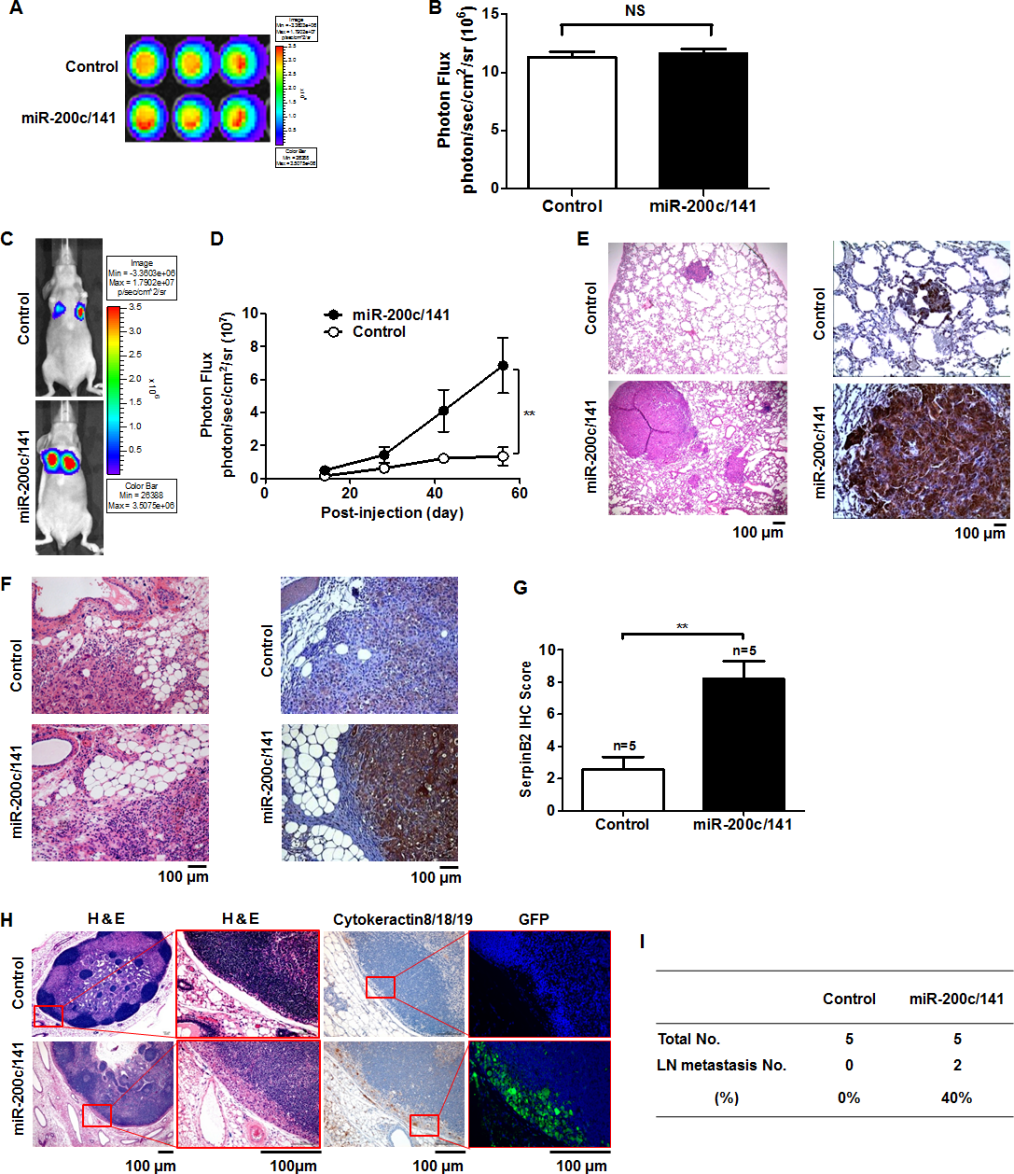
miR-200c/141 cluster overexpression increases lung and LN metastasis in a mouse xenograft model Representative BLI of control and MDA-MB-231^miR-200c/141^ cells **(A)** Total photon flux of the bioluminescent signals emitted from control and MDA-MB-231^miR-200c/141^ cells. **(B)** Representative BLI of lung metastasis mice at 58 days **(C)** Total photon flux of the bioluminescent signals emitted from control and MDA-MB-231^miR-200c/141^ cell xenografted mouse thorax regions 58 days post-injection **(D)** Representative H&E and SerpinB2 immunostaining images in lung and primary tumor tissues **(E** & **F)** SerpinB2 IHC scores from control and MDA-MB-231^miR-200c/141^ mouse primary tumors **(G)** Representative H&E and cytokeratin8/18/19, GFP, and SerpinB2 immunostaining images in regional LN metastases distant from the primary tumor **(H)** Regional LN metastasis investigated in control and MDA-MB-231^miR-200c/141^ mice **(I)** **P<0.01. NS, not significant.

Figure 2A shows bioluminescence imaging (BLI) of controls and MDA-MB-231^miR-200c/141^ cells. Total photon fluxes analyzed by measuring bioluminescent signals emitted from controls and MDA-MB-231^miR-200c/141^ cells were 11.30×10^6^ ± 0.49 photon/sec/cm^2^/sr and 11.60×10^6^ ± 0.44 photon/sec/cm^2^/sr, respectively (Figure 2B). Within two weeks post-intravenous injection, localized bioluminescent signals indicating lung metastasis began to appear at the thorax in each mouse (Figure 2C). At 56 days, these signals were up to 5-fold greater in MDA-MB-231^miR-200c/141^ mice (6.85×10^7^ ± 16.85 photon/sec/cm^2^/sr) than in controls (1.34×10^7^ ± 5.05 photon/sec/cm^2^/sr) (Figure 2D, *P*=0.007). Lung metastases were greater in number and mass in MDA-MB-231^miR-200c/141^ tumors compared to controls, and SerpinB2 overexpression was observed in MDA-MB-231^miR-200c/141^ tumor lung metastases (Figure 2E). SerpinB2 expression was stronger in orthotopic primary tumors of MDA-MB-231^miR-200c/141^ mice compared to controls at 40 days (Figures 2F and 2G, P=0.003). Cytokeratin 8/18/19, GFP and SerpinB2 immunostaining showed LN metastases only in MDA-MB-231^miR-200c/141^ tumors, and not in controls (Figure 2H). Regional LN metastasis distant from the primary tumor was observed in two cases of five MDA-MB-231^miR-200c/141^ mice, while LN metastasis was not detected in controls (Figure 2I).

### SerpinB2 knockdown suppresses miR-200c/141 cluster overexpression-induced lung metastasis in mice

**Figure 3:**
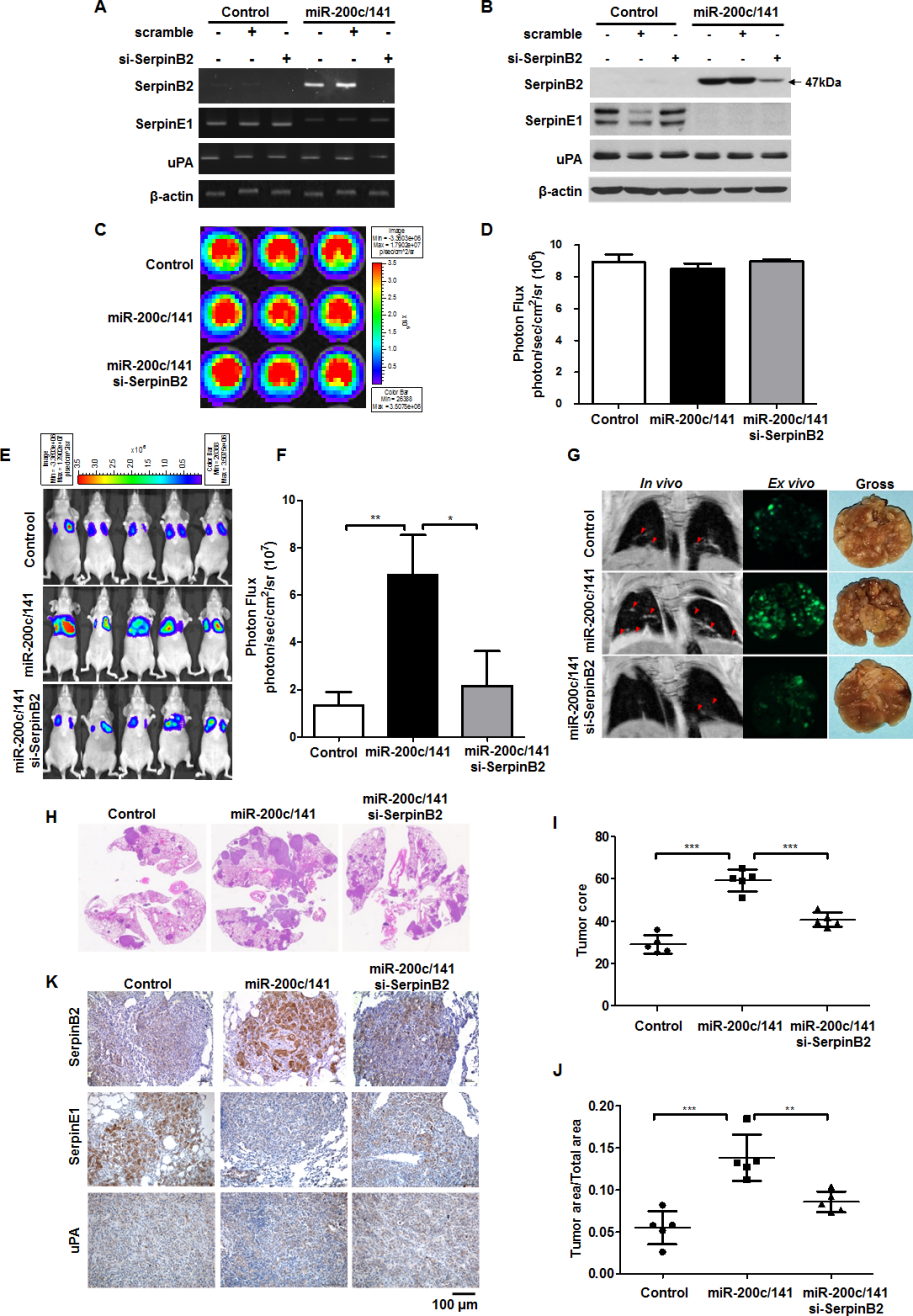
SerpinB2 knockdown decreased lung metastasis promoted by miR-200c/141 overexpression in BC cells Representative RT-PCR **(A)** and western blotting results **(B)** for SerpinB2, SerpinE1, and uPA in control and MDA-MB-231^miR-200c/141^ cells transfected with SerpinB2 or scramble siRNA. Representative BLI **(C)** and total photon flux **(D)** of the bioluminescent signals in control, MDA-MB-231^miR-200c/141^, and MDA-MB-231^miR-200c/141^+si-SerpinB2 cells. Representative BLI of mice injected with control, MDA-MB-231^miR-200c/141^, or MDA-MB-231^miR-200c/141^+si-SerpinB2 cells **(E)** Total photon flux of the bioluminescent signals emitted from thorax regions of control, MDA-MB-231^miR-200c/141^, and MDA-MB-231^miR-200c/141^+si-SerpinB2 mice **(F)** Representative *in vivo* MRI (red arrowheads), *ex vivo* GFP fluorescence, and gross images of lung metastatic nodules from control, MDA-MB-231^miR-200c/141^, and MDA-MB-231^miR-200c/141^+si-SerpinB2 mice **(G)** Representative H&E staining in lung tissues **(H)** Tumor score **(I)** and area **(J)** of individual lung metastases evaluated via H&E staining in control, MDA-MB-231^miR-200c/141^, and MDA-MB-231^miR-200c/141^+si-SerpinB2 mice. Representative SerpinB2, SerpinE1, and uPA immunostaining in lung tissues **(K)** **P<0.01; ***P<0.001.

miR-200c/141 overexpression upregulated SerpinB2, and siRNA-mediated SerpinB2 knockdown successfully reduced SerpinB2 levels in MDA-MB-231^miR-200c/141^ cells (Figures 3A and 3B). SerpinB2 knockdown did not affect SerpinE1 or uPA levels. 5×10^3^ control cells, MDA-MB-231^miR-200c/141^+scramble cells and MDA-MB-231^miR-200c/141^+si-SerpinB2 cells exhibited 8.9×10^6^ ± 0.7 photon/sec/cm^2^/sr, 8.9×10^6^ ± 0.4 photon/sec/cm^2^/sr, and 9.0×10^6^ ± 0.2 photon/sec/cm^2^/sr, respectively (Figure 3C and 3D). A wound-healing assay showed that SerpinB2 knockdown decreased MDA-MB-231^miR-200c/141^ cell migration (Supplementary Figure 4).

At 56 days, strong bioluminescent signals were observed in the lungs of MDA-MB-231^miR-200c/141^ mice compared with controls, but signals were decreased in MDA-MB-231^miR-200c/141^+si-SerpinB2 mice (2.17×10^7^ ± 14.64 photon/sec/cm^2^/sr) (Figures 3E and 3F, P=0.035). Consistent with BLI and magnetic resonance imaging (MRI), GFP imaging and gross anatomy revealed that miR-200c/141 overexpression promoted increased numbers and masses of MDA-MB-231 cell lung metastases, whereas SerpinB2 knockdown suppressed MDA-MB-231^miR-200c/141^ cell metastasis (Figure 3G). H&E staining of lung sections (Figure 3H) showed that lung metastasis scores and areas were greater in MDA-MB-231^miR-200c/141^ than in control (*P*<0.001), and were reduced in MDA-MB-231^miR-200c/141^+si-SerpinB2 (*P*<0.001 and *P*=0.0045) (Figures 3I and 3J). Lung metastatic tumor tissue immunohistochemistry (IHC) analyses revealed that miR-200c/141 overexpression upregulated SerpinB2 in cancer cells, but downregulated SerpinE1 (Figure 3K).

### SerpinB2 protein is overexpressed in the TN subtype relative to other subtypes

We investigated relationships between SerpinB2 expression and clinicopathological features (Table 1). High SerpinB2 expression was detected in 216 cases (64.1%), and low expression was found in 121 cases (35.9%). SerpinB2 was more likely to be overexpressed in tumors ≥2 cm in size (69.2%) compared with tumors <2 cm (52.0%; *P*<0.01). With regard to TNM clinical stage, SerpinB2 was overexpressed in 76.9% of advanced stage (III–IV) cases, but only 58.1% of early stage (I–II) cases (*P*<0.001). 75.0% of cases with LN metastasis had high SerpinB2 levels, as compared to only 55.6% of cases with no LN metastasis (*P*<0.001).

**Table 1:** Correlation between SerpinB2 overexpression and BC clinical parameters

Parameters	No. of cases	SerpinB2-high (%)	SerpinB2-low (%)	*P*-value
**Breast cancer**	337	216(64.1)	121(35.9)	
**Age**				
≧50	235	156(66.4)	79(33.6)	0.184
<50	102	60(58.8)	42(41.2)	
**Tumor size**				
≧2cm	237	164(69.2)	73(30.8)	0.002
<2cm	100	52(52.0)	48(48.0)	
**TNM stage**				
I-II	229	133(58.1)	96(41.9)	0.000
III-IV	108	83(76.9)	25(23.1)	
**Histology grade**				
Well	59	34(57.6)	25(42.4)	0.129
Moderate	206	129(62.6)	77(37.4)	
Poor	72	53(73.6)	19(26.4)	
**LN metastasis**				
Negative	189	105(55.6)	84(44.4)	0.000
Positive	148	111(75.0)	37(25.0)	
**ER**				
Negative	144	97(67.4)	47(32.6)	0.2803
Positive	193	119(61.7)	74(38.3)	
**PR**				
Negative	170	111(65.3)	59(34.7)	0.643
Positive	167	105(62.9)	62(37.1)	
**HER2**				
Negative	192	125(65.1)	67(34.9)	0.657
Positive	145	91(62.8)	54(37.2)	

ER, estrogen receptor; PR, progesterone receptor; HER2, human epidermal receptor 2.

**Figure 4:**
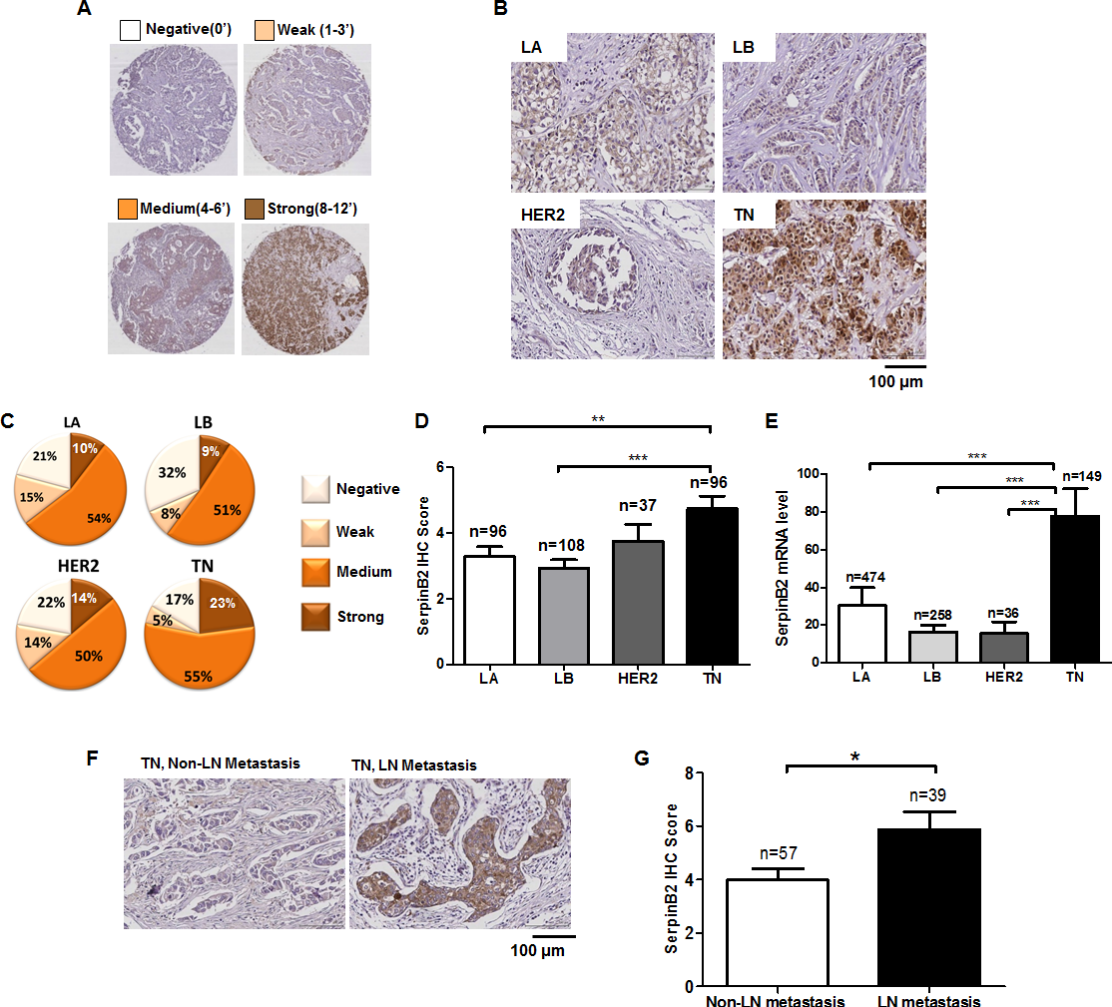
SerpinB2 is overexpressed in TNBC relative to other BC subtypes Semi-quantitative SerpinB2 IHC score evaluated via staining intensity and the rate of positive cancer cells in primary BC tissues **(A)** Representative SerpinB2 immunostaining in four primary BC subtypes, LA, LB, HER2, and TN **(B)** Cancer cell populations according to SerpinB2 IHC score (negative, week, medium, strong) in LA, LB, HER2, and TN subtypes **(C)** SerpinB2 IHC score in LA, LB, HER2, and TN subtypes **(D)** SerpinB2 levels evaluated from BC subtypes (LA, n=474; LB, n=256; HER2, n=36; and TN, n=149) using RNASeqV2 data **(E**) Representative SerpinB2 immunostaining in TNBC tissues with or without LN metastasis **(F)** SerpinB2 IHC scores of TNBC tissues with or without LN metastasis **(G)** *P<0.05; **P<0.01; ***P<0.001.

Figure 4A shows SerpinB2 IHC scores based on a predetermined cutoff. TN subtypes exhibited strong SerpinB2 expression (Figures 4B and 4C). SerpinB2 IHC scores were higher in the TN subtype (4.75 ± 0.38) than in the LA (3.29 ± 0.29) or LB (2.93 ± 0.26) subtypes, but not the HER2 subtype (3.76 ± 0.51) (LA vs. TN, *P*=0.0027, LB vs. TN, *P*<0.0001, LB vs. TN, *P*=0.1535; Figure 4D). TCGA data analysis confirmed that SerpinB2 expression was highest in the TN subtype (Figure 4E).

High SerpinB2 expression was observed in the TN subtype with LN metastasis, but SerpinB2 was only rarely detected (and at low levels) in the TN subtype without LN metastasis (Figures 4F and 4G). In xenografts and TN patient tumor samples, high SerpinB2 expression was also observed in macrophages and fibroblasts in adjacent stromal tissue (Supplementary Figure 5). MDA-MB-231^miR-200c/141^ cell xenograft tumors displayed extensive macrophage infiltration (stained with F4/80 as a pan macrophage marker) into lung tissues, and SerpinB2 knockdown decreased macrophage infiltration into these tumors (Supplementary Figure 6).

### SerpinE1 and uPA levels are the same in LA, LB, HER2, and TN subtypes

**Figure 5:**
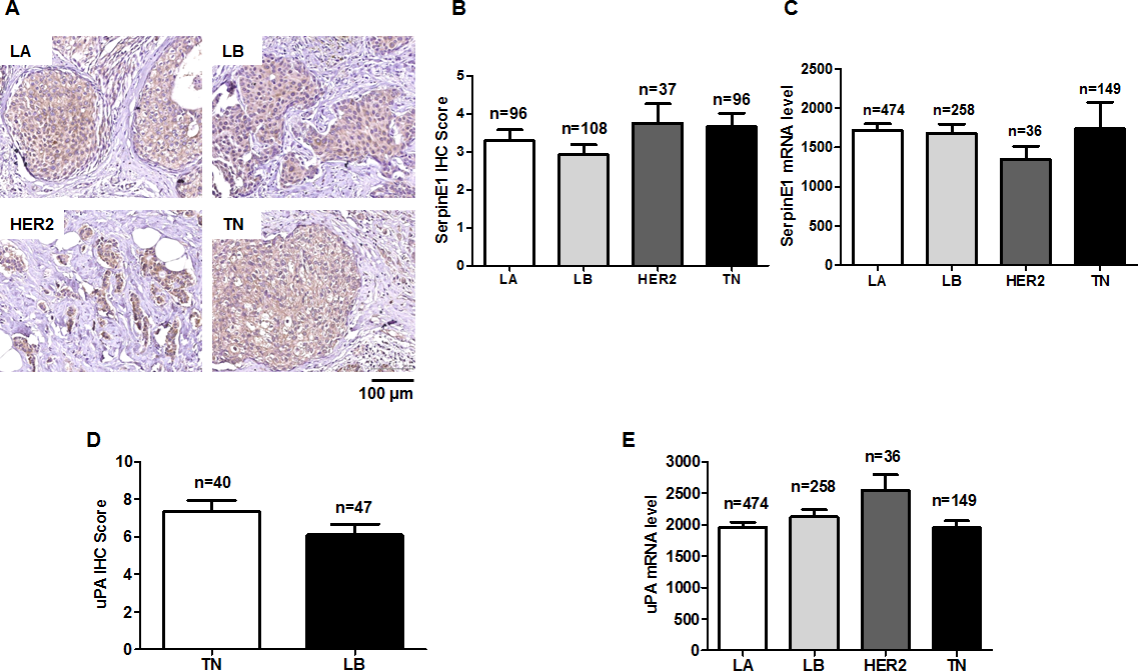
SerpinE1 and uPA levels do not differ among LA, LB, HER2, and TN subtypes Representative SerpinE1 immunostaining in primary BC tissues of LA, LB, HER2, and TN subtypes **(A)** SerpinE1 IHC scores in LA (n=96), LB (n=108), HER2 (n=37), and TN (n=96) subtypes **(B)** SerpinE1 levels in LA (n=474), LB (n=256), HER2 (n=36), and TN (n=149) subtypes using RNASeqV2 data **(C)** uPA IHC scores in LB (n=47) and TN (n=40) subtypes **(D)** uPA levels in LA (n=474), LB (n=256), HER2 (n=36), and TN (n=149) subtypes using RNASeqV2 data **(E)**.

Consistent with TCGA data analyses, SerpinE1 staining did not differ among LA (3.29 ± 0.29), LB (2.93 ± 0.26), HER2 (3.76 ± 0.51) and TN (3.67 ± 0.35) subtypes (Figures 5A, 5B and 5C). A slightly increased uPA IHC score was observed in the TN subtype relative to the LB subtype, but was not significant (Figure 5D). uPA levels in the TCGA data analysis did not different among the four subtypes (Figure 5E).

### SerpinB2 overexpression is associated with reduced overall survival (OS) in BC patients

**Figure 6:**
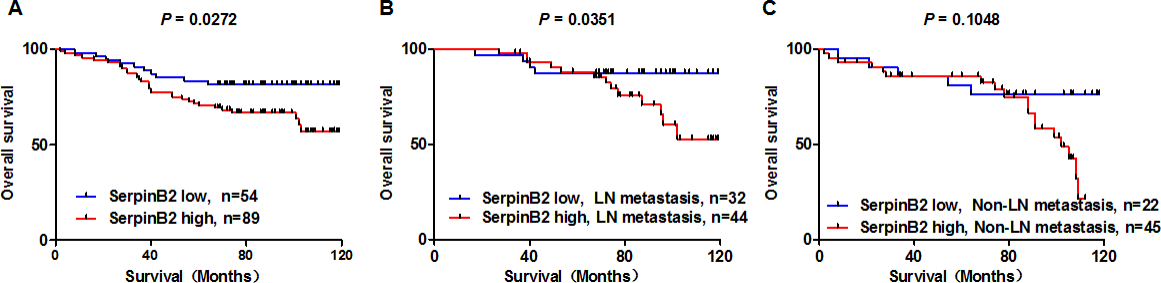
SerpinB2 overexpression is associated with unfavorable BC patient survival outcomes OS analysis by high or low SerpinB2 protein level in patient primary tumors **(A)** OS analysis in high and low SerpinB2-expressing cases in patients with **(B)** or without **(C)** LN metastasis.

Median OS in 143 BC patients was 77.59 months. OS was reduced for patients with SerpinB2-overexpressing tumors (Figure 6A, *P*=0.027). SerpinB2 overexpression was also associated with reduced OS in patients with LN metastasis (Figure 6B, *P*=0.035), but not in those without LN metastasis (Figure 6C, *P*=0.105).

In a univariate analysis, patients with high SerpinB2 expression had reduced OS compared to patients with low expression (HR: 1.410, 95% CI: 1.000; *P*=0.05). LN metastasis (HR: 3.40, 95% CI: 2.326–4.85; *P*=0.000), TNM stage (HR: 1.856, 95% CI: 1.31–2.621; *P*=0.000) and HER2 status (HR: 2.590, 95% CI: 1.810–3.705; *P*=0.000) were associated with OS (Table 2). Multivariate analysis was performed using the Cox proportional hazard model for all significant variables examined in the above univariate analysis. LN metastasis (HR: 3.014, 95% CI: 1.884–4.823; *P*=0.000) and HER2 status (HR: 2.474, 95% CI: 1.688–3.625; *P*=0.00) were significant in the multivariate analyses.

**Table 2:** Univariate and multivariate survival analyses (Cox regression model) of various factors in BC patients

					95.0% CI for Exp(B)		
	B	SE	Wald	HR	Lower	Upper	*P*-value
**Univariate analysis**							
SerpinB2	0.341	0.174	3.833	1.406	1.000	1.978	0.050
Age	0.063	0.172	0.133	1.065	0.760	1.491	0.715
Histology grade	−0.058	0.170	0.116	0.944	0.676	1.317	0.733
Size	0.370	0.192	3.720	1.448	0.994	2.109	0.054
Lymph node	1.212	0.188	41.725	3.360	2.326	4.853	0.000
TNM Stage	0.619	0.176	12.353	1.856	1.315	2.621	0.0004
ER	0.108	0.169	0.406	1.114	0.799	1.553	0.524
PR	0.209	0.185	1.282	1.233	0.858	1.771	0.258
HER2	0.952	0.183	27.133	2.590	1.810	3.705	0.000
**Multivariate analysis**							
SerpinB2	0.166	0.189	0.772	1.181	0.815	1.710	0.380
Age	−0.128	0.191	0.448	0.880	0.605	1.279	0.503
Histology grade	−0.091	0.181	0.251	0.913	0.641	1.302	0.616
Size	−0.035	0.204	0.029	0.966	0.647	1.442	0.866
Lymph node	1.103	0.240	21.159	3.014	1.884	4.823	0.000
TNM Stage	0.019	0.235	0.006	1.019	0.642	1.617	0.936
ER	0.201	0.238	0.714	1.223	0.767	1.949	0.398
PR	−0.101	0.263	0.147	0.904	0.540	1.514	0.701
HER2	0.906	0.195	21.593	2.474	1.688	3.625	0.000

B, coefficient of regression; SE, standard error; Wald, chi-square, HR, hazard ratio; CI, confidence interval; ER, estrogen receptor; PR, progesterone receptor; HER2, human epidermal receptor 2.

We evaluated co-expression relationships between miR-200c, miR-141, and SerpinB2 in TNBC patient primary tumors (n=19). miR-200c (*P*=0.098) and miR-141 (*P*=0.013) levels positively correlated with SerpinB2 mRNA (Supplementary Figure 7). However, miR-200c/141 expression was not associated with TNBC patient clinicopathological features (Supplementary Table 1).

## DISCUSSION

In BC patients and animal models, high miR-200c and miR-141 levels have been associated with enhanced metastatic colonization and poor clinical outcomes [7, 9, 10, 22, 23]. Consistent with these observations, we found that stable miR-200c/141 cluster overexpression in TNBC MDA-MB-231 cells promoted lung metastasis in a mouse model. miR-200 members regulate global gene expression through RNA silencing and direct or indirect post-transcriptional targeting, thereby modulating many biological processes, including cell cycle control, proliferation, apoptosis, and invasion [1, 2, 4–8, 23, 24]. Here, we observed altered gene expression in MDA-MB-231 cells overexpressing miR-200c/141, with 10 genes (SerpinB2, MAL2, C15orf54, PLCβ4, MPZL2, LCP1, KRTAP2-4, EDN1, ID2, EGR1) upregulated. These genes were not predicted targets of miR-200c/141 cluster members. We also found that miR-200c/141 overexpression increased expression and nuclear localization of SerpinB2 transcription factors (c-Jun, FosB and c-Fos), increased SerpinB2 promoter activity, and downregulated miR-26b and miR-124a, suggesting that miR-200c/141 directly or indirectly affects transcription factors and/or miRNAs to upregulate SeripinB2.

SerpinB2 is upregulated during inflammation, and is expressed in diverse cell types, including cancer cells, macrophages, and fibroblasts [13]. While SerpinB2 has been studied with respect to extracellular matrix remodeling, tumor growth, and metastasis in diverse cancers, its association with patient prognosis is unclear [16, 18, 20, 21, 25, 26]. SerpinB2 exists in two forms, an extracellular form (>60 kDa) and a predominant intracellular form (47 kDa), which can be upregulated by inflammation or stress-related factors, and released into the extracellular space [13, 14]. Secreted SerpinB2 inhibits extracellular uPA, which is associated pericellular and extracellular proteolysis and intracellular SerpinB2 interacts with a variety of cytosolic proteins, which is involved in intracellular signaling pathways to regulate cell survival, but its intracellular activities are not yet well-understood [15]. SerpinB2 also defends against tumoral invasion by preventing plasminogen activation [16, 26].

TNBC molecular subtypes were subclassified as two basal-like (BL1 and BL2), an immunomodulatory (IM), a mesenchymal (M), a mesenchymal stem-like (MSL), and a luminal AR (LAR) [27]. In the present study, SerpinB2 expression level was investigated in carcinosarcoma-derived Hs578T cell (MSL subtype), ductal carcinoma-derived HCC-38 cell (BL1 subtype) and invasive ductal carcinoma-derived MDA-MB-231 cell (MSL subtype). The stable overexpression of the miR-200c/141 cluster increased both SerpinB2 forms, and decreased SerpinE1 in MDA-MB-231 cells. Secreted SerpinB2 was predominantly in its >60 kDa form in CM from both MDA-MB-231^miR-200c/141^ and control cells, and a high amount of the 47-kDa form was observed in MDA-MB-231^miR-200c/141^ cell CM. On the contrary to the effect of miR-200c/141 on increased SerpinB2 expression in MDA-MB-231 cells, SerpinB2 mRNA and protein were downregulated in Hs578T^miR-200c/141^ and HCC-38^miR-200c/141^ cells. Based on our results, the effect of miR-200c/141 on SerpinB2 expression is only MDA-MB-231 cell type specific. Our data suggest that miR-200c/141 cluster overexpression is likely responsible for SerpinB2 upregulation and release in MDA-MB-231 cells.

The c-Jun, c-Fos and FosB which are SerpinB2 transcription factors [28] increased in MDA-MB-231^miR-200c/141^ cells compared to control. miR-124a and miR-26b which are able to target SepinB2 [29] decreased in MDA-MB-231^miR-200c/141^ cells compared to control. miRNAs have the capability of activating gene expression through post-transcriptional regulation for example, increased mRNA stability and miRNA-mediated decoy [30]. There is, as yet, no report of underlying mechanism by which miR-200c/141 increase c-Jun, c-Fos, and FosB or decrease miR-124a and miR-26b. Del Vecchio G *et al*. reported that miR-200a could increases the c-Jun amount through a microRNA-mediated stabilization of its mRNA [31]. A common mechanism for miR-124 downregulation is the methylation of the promoter region of its gene through transcriptional regulators [32]. We speculate that miR200c/141 may involve in posttranscriptional regulation for upregulation of c-Jun and c-Fos via miRNA-mediated mRNA stability and miRNA-mediated decoy. Decreases of miR-124a and miR-26b in MDA-MB-231^miR-200c/141^ cells may be due to the overexpression of genes to regulate hypermethylation. We will further investigate underlying mechanisms by which miR-200c/141 increases c-Jun and c-Fos and decreases miR-124a and miR-26b.

With respect to the role of SerpinB2 in lung metastasis, we demonstrated that SerpinB2 knockdown suppressed miR-200c/141 overexpression-induced migration and lung metastasis by MDA-MB-231 cells. Mouse xenograft results showed that upregulation of SerpinB2 by miR-200c/141 overexpression might be a main mechanism for increased lung metastasis in MDA-MB-231 cells. Thus, SerpinB2 likely represents an effector or mediator of miR-200c/141 cluster-induced metastatic behavior in TNBCs. Recently, SerpinB2 in BCCs was shown to promote cancer cell survival and metastatic outgrowth by converting astrocytic FasL into a paracrine death signal for cancer cells, and by inactivating L1 cell adhesion molecule (L1CAM) to spread metastatic cells along brain capillaries [21]. Many studies have linked SerpinB2 to macrophage survival as well as the tumor-associated M2 phenotype in lung metastasis [21, 33–35]. We observed macrophage infiltration within lung metastatic foci of MDA-MB-231^miR-200c/141^ xenograft tumors; SerpinB2 knockdown decreased this infiltration, suggesting that SerpinB2 promotes TNBC cell lung metastasis via macrophages recruitmentto tumors.

Overexpression of plasminogen activation system members, including uPA, SerpinE1 and SerpinB2, is associated with malignancy, tumor progression, and metastasis [17, 19]. SerpinB2 inhibition of uPA in tumors is of significant interest in prognosis and clinical outcome prediction in various cancers [16, 20, 21, 36]. Although others have explored the prognostic value of SerpinB2 in BC patients, the status of SerpinB2 from tumor extracts was evaluated using RT-PCR and immunoenzymatic assays, which do not discriminate between cancer and stromal cells that also express SerpinB2 [16, 20, 36–40]. However, IHC analysis can reveal specific SerpinB2 locations in tumor tissues. The present study incorporated TCGA datasets, the University of North Carolina's RNASeqV2, and IHC score analysis for SerpinE1, SerpinB2 and uPA. We observed high SerpinB2 levels in TNBC relative to other BC subtypes, and SerpinE1 and uPA levels differed between LA, LB, HER2, and TN subtypes. Additionally, SerpinB2 expression was higher in primary tumors with LN metastasis than in non-LN metastasis cases, indicating that high SerpinB2 status may be a prognostic factor for predicting LN metastasis.

Bouchet, *et al*. and Foekens, *et al*. suggest that SerpinB2 overexpression may be a marker for prolonged survival in BC patients with low uPA and SerpinE1 levels [26, 36]. They evaluated SerpinB2 levels from primary tumor lysates using immunoenzymatic assays, which do not reflect the expression site of SerpinB2 in tumor cells. In contrast to these studies, our IHC results showed that high levels of SerpinB2 in cancer cells within the primary tumor tissue were associated with reduced OS for all BC patients. In our study, tumor cells overexpressing SerpinB2 did not display SerpinE1 or uPA downregulation. Our results suggest SerpinB2 overexpression in BCCs with concomitant SerpinEl or uPA expression might contribute to poor prognosis and unfavorable clinical outcomes. Univariate analysis confirmed that a poor outcome was associated with SerpinB2 status, HER2, TNM stage, and LN metastasis in all BC patients. In multivariate analysis, SerpinB2 is not independent prognostic factor, but contribute to LN status or HER2 expression associated with poor outcome.

Korpal M, *et al*. reported high miR-200 family levels in lung-pleural metastases with reduced BC patient survival and poor prognosis [7]. This is consistent with our xenograft results, and supports the potential role of the miR-200 family in promoting metastasis. However, to our knowledge our study was the first to investigate relationships between miR-200c, miR-141, and SerpinB2 in BC tumor tissues. In TNBC patients, we found a positive correlation between miR-200c, miR-141 and SerpinB2 mRNA. Together, our findings suggest that miR-200c, miR-141 and SerpinB2 are poor prognostic factors in TNBC.

In conclusion, SerpinB2 upregulation was shown to be involved in miR-200c/141 cluster-promoted lung and LN metastasis in BC xenograft models. In patient tumor samples, higher SerpinB2 levels were detected in TNBC as compared to other BC subtypes, and were associated with LN metastasis and reduced survival. Taken together, our findings provide new insights into the role of SerpinB2, which was upregulated by miR-200c/141 overexpression, in promoting BCC lung and LN metastasis, and suggest that SerpinB2 could be used to assess metastasis risk in BC patients.

## MATERIALS AND METHODS

### Cell culture and lentiviral transduction

TNBC MDA-MB-231 cells were purchased from the Korean Cell Line Bank. Cells were cultured in RPMI-1640 (WelGENE, Seoul, Korea) containing 10% fetal bovine serum (FBS), 2 mM L-glutamine, and 1% penicillin/streptomycin, grown in a 5% CO_2_ incubator at 37°C, and subcultured weekly. Lentiviral vectors containing the miR-200c/141 cluster (GenBank ID: 406985 406933) and GFP constructs were kindly supplied by Dr. Gregory J. Goodall [41]. After transduction of miR-200c/141-GFP lentivirus into MDA-MB-231 cells and selection with puromycin (3 μg/ml), MDA-MB-231 cells overexpressed miR-200c/141 were sorted using a FACSCalibur flow cytometer (BD Biosciences, San Joese, CA, USA). Additionally, lentivirus containing firefly luciferase and GFP was transduced into miR-200c/141-overexpressing MDA-MB-231 cells for animal studies. Cells stably expressing firefly luciferase were selected with zeocin (50 μg/ml). MDA-MB-231 and miR-200c/141-overexpressing MDA-MB-231 cells are referred to as control and MDA-MB-231^miR-200c/141^.

### Microarray analysis and quantitative real-time RT-PCR

Gene expression profiling was performed using the Human Gene 1.0 STmicroarray (Affymetrix, Santa Clara, CA, USA) with control and MDA-MB-231^miR-200c/141^ cells. Only genes with *P*≤0.05 and fold change ≥ 1.5 were considered for further analysis. Specific primers for SerpinB2, SerpinE1, uPA, MAL2, C15orf54, PLCβ4, MPZL2, LCP1, KRTAP2-4, EDN1, ID2, EGR1, c-Jun, c-Fos, and FosB and β-actin are provided in Supplementary Table 2. Real-time PCR reactions were run on an ABI PRISM® 7900 using a SYBR Green PCR master mix (Applied Biosystems, Foster City, CA, USA). Results were analyzed by the ΔCt method, which reflects the threshold difference between a target gene and β-actin in each sample. TaqMan MicroRNA Assays (Applied Biosystems, South San Francisco, CA, USA) were used to quantify mature miRNA levels, following the manufacturer's instructions. miRNAs were isolated from cells using the mirVana miRNA isolation kit (Applied Biosystems), and primers for detecting miR-200c, miR-141, miR-124a and miR-26b were purchased from Applied Biosystems. Reverse transcription was performed using the TaqMan microRNA reverse transcription kit (Applied Biosystems) according to the manufacturer's instructions. RUN48 was used as the endogenous control.

### Chloramphenicol acetyltransferase (CAT) reporter assay

Cells (1×107) were transfected with 20 μg of plasmid reporter DNA using Lipofectamine-2000 (Thermo Fisher Scientific, Inc.). CAT assays were performed with 50 μg of whole cell lysate using the CAT reporter gene activity detection kit (Sigma-Aldrich). SerpinB2 promoter-mediated CAT activity was normalized to the positive control plasmid, pCAT-Control (Promega), to allow comparison of the results of independent experiments.

### siRNA transfection

The siRNA duplexes designed to knockdown SerpinB2 expression were purchased from Bioneer. siRNA sequences were as follows: 1) SerpinB2 siRNA sense, AAGGUAUCCCUAUUUUUGAAGCCUGU CUC and antisense, AACUUCAAAAAUAGGGA UACCCCUGUCUC 2) SerpinB2 siRNA sense, GAGCUUCCGGGAAGAAUAU and antisense, AUAUUCUUCCCGGAAGCUC 3) SerpinB2 siRNA sense, CAGAGAACAACCAGAUUGA and antisense, UCAAUCUGGUUGUUCUCUG. Scramble siRNA (AccuTarget™ Negative Control siRNA, BioRP) (Bioneer, Daejeon, Korea) was also used. Cells were transfected with SerpinB2 siRNA or scramble siRNA using Lipofectamine-2000 (Thermo Fisher Scientific Inc, Carlsbad, CA, USA).

### Western blot analysis

Proteins from cells lysed in RIPA buffer were separated using SDS-PAGE and transferred to nitrocellulose membranes. Membranes were blocked using 5% skim milk in Tris-buffered saline containing Tween-20, incubated overnight at 4°C with primary antibodies directed against SerpinB2 (Abcam, Cambridge, UK), SerpinE1 (Abcam), uPA (Abcam), c-Jun (Santa Cruz Biotechnology, Santa Cruz, CA, USA) or phospho-c-Jun (Santa Cruz Biotechnology), and β-actin (Sigma-Aldrich, St. Louis, MA, USA), and then incubated with HRP-conjugated secondary antibodies (Santa Cruz Biotechnology). Blotted membranes were visualized using enhanced chemiluminescence reagents (GE Healthcare, Piscataway, NJ, USA) and protein expression was depicted as intensity of each protein relative to that of β-actin using ImageJ software.

### Xenograft animal model

All animal experiments were approved by the Seoul National University Hospital Biomedical Research Institute Animal Care and Use Committee (IACUC). A total of 28 female BALB/c nude mice were used. Orthotopic xenografts were established via injection of 1×10^6^ MDA-MB-231^miR-200c/141^ (n=5) or control cells (n=5) into the fat pad of the 4th mammary gland of 5-week old mice. To investigate lung metastasis, 5×10^5^ cells transfected additionally with SerpinB2 or scramble siRNA were suspended in 0.1 ml of PBS and injected into the tail vein of 6-week old mice. Tumor-bearing mice were randomly assigned to one of three groups: MDA-MB-231 (n=6); MDA-MB-231^miR-200c/141^ (n=6); and MDA-MB-231^miR-200c/141^+si-SerpinB2 (n=6).

### Bioluminescence imaging (BLI)

BLI was conducted using the IVIS luminal II system (Caliper Life Sciences, Hopkinton, MA, USA) with living image acquisition and analysis software. After administration of D-luciferin (150 μg/ml), bioluminescent signal in 1×10^3^ to 1×10^5^ cells per well was quantified. Mice were intraperitoneally injected with D-luciferin (150 mg/kg). BLI of mice was performed weekly post-injection, and the bioluminescent signal of the thorax area was quantified.

### Magnetic resonance imaging (MRI) and fluorescence imaging

At 58 days post-injection, *in vivo* MRI studies were performed on a 9.4 Tesla animal MR scanner (Agilent Technologies, Palo Alto, CA, USA) using a one-channel volume Millipede Coil. *In vivo* T_2_-weighted mouse thorax images were acquired in the coronal plane using fast spin echo sequence. *Ex vivo* GFP fluorescence images of excised lungs were obtained and analyzed using Maestro imaging system (CRI Inc, Woburn, MA, USA).

### Clinical samples and ethics statement

A total of 480 tissue microarrays of primary tumors in patients who were not treated before surgery, along with medical records, were collected from Outdo Biotech Co. Ltd. (Shanghai, China) between Aug 2004 and Dec 2008. BC subtypes were defined as luminal A (LA, n=96), luminal B (LB, n=108), HER2 (n=37) and TN (n=96), regarding ER, PR and HER2 status. Follow-up survival data collected from 143 patients for more than 10 years were used. The follow-up deadline was Jul 2014, at which point 42 patients had died, and 101 remained alive. Survival time was counted from the date of surgery to the follow-up deadline or date of death (all patients eventually died of cancer recurrence or metastasis). Total RNAs were isolated from breast cancer tissue of 21 patients with TNBC and real-time RT-PCR was performed to analyze miR-200c, miR-141 and SerpinB2 mRNA. The Institutional Review Board (IRB) of Seoul National University Hospital (IRB No. 1604-063-754 and 1608-131-787) reviewed and approved the study protocol, and exempted the study from the obligation to obtain informed consent. This study was performed in observance of the World Medical Association's Declaration of Helsinki.

### Histological analysis

Tissues were fixed with 10% buffered formalin and embedded in paraffin blocks. Routine H&E staining and immunostaining of SerpinB2, SerpinE1, uPA, cytokeratin8/18/19, and GFPwas performed. Primary SerpinB2, SerpinE1, uPA, cytokeratin8/18/19 (Abcam), GFP(Santa Cruz Biotechnology) and F4/80 (Thermo Fisher Scientific Inc) antibodies, and secondary HRP-conjugated antibodies (DAKO, Carpinteria, CA, USA) or Alexa Fluor® 488 (Thermo Fisher Scientific Inc) were used. Semi-quantitative IHC scores were interpreted according to a combination of staining intensity and rate of positive cancer cells. Negative (0) and weak (1–3) were included in the low-expression group, with medium (4–6) and strong (8–12) in the high-expression group.

### Analysis of the cancer genome atlas (TCGA) dataset

From the TCGA data portal, we downloaded clinical information and RNA-seq expression profiles for 917 primary BC tumors. For RNA-seq expression profiles, we used normalized Level-3 gene expression data produced by University of North Carolina's RNASeqV2 pipeline using the Illumina HiSeq platform. Based on the clinical information, average expression values were calculated for SerpinB2, SerpinE1, and uPA in LA (n=474), LB (n=258), HER2 (n=36) and TN (n=149) subtypes.

### Statistical analyses

For both *in vitro* and *in vivo* data, means ± standard deviations were calculated from at least five independent experiments and statistical analysis of data was performed using analysis of variance (ANOVA) and Student-Newman-Keuls test. For clinical data analysis, the Chi-square test was used to compare baseline characteristic distributions among groups. Survival analyses were performed using the Kaplan Meier method. Cox proportional hazard regression models were used to estimate hazard ratios (HR) with 95% confidence intervals (CI). A multivariate Cox regression model was fitted based on all characteristics that had *P*≤0.10 in the univariate analysis. For all tests, a *P*<0.05 was considered significant.

## SUPPLEMENTARY MATERIALS FIGURES AND TABLES


