# Supplementary material for: microRNA-200c/141 upregulates SerpinB2 to promote breast cancer cell metastasis and reduce patient survival
Source: Oncotarget. 2017 Feb 24;8(20):32769–82. doi: 10.18632/oncotarget.15680 (PMC5464826; doi:10.18632/oncotarget.15680)
Supplement: Supplementary file 1 [file oncotarget-08-32769-s001.pdf]

microRNA-200c/141 upregulates SerpinB2 to promote breast cancer cell metastasis and reduce patient survival

SUPPLEMENTARY FIGURES AND TABLES

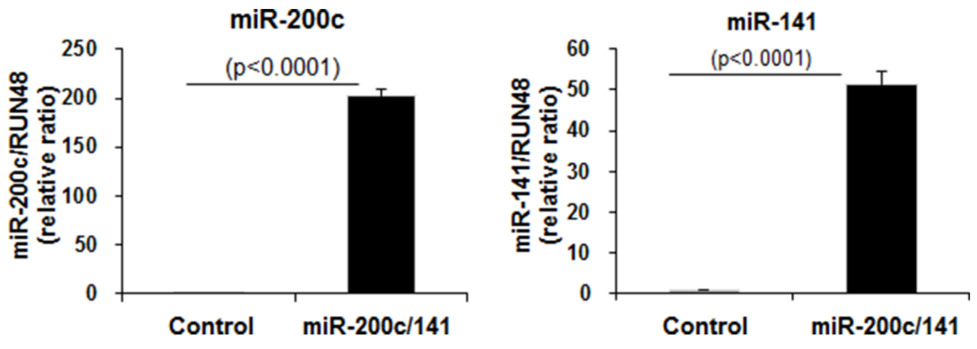

Supplementary Figure 1: Real-time RT-PCR analysis of miR-200c and miR-141 in MDA-MB-231$^{miR\text{-}200c/141}$ cells. miR-200c and miR-141 levels were significantly higher in MDA-MB-231$^{miR\text{-}200c/141}$ cells relative to control (n=3). ****P*<0.0001.

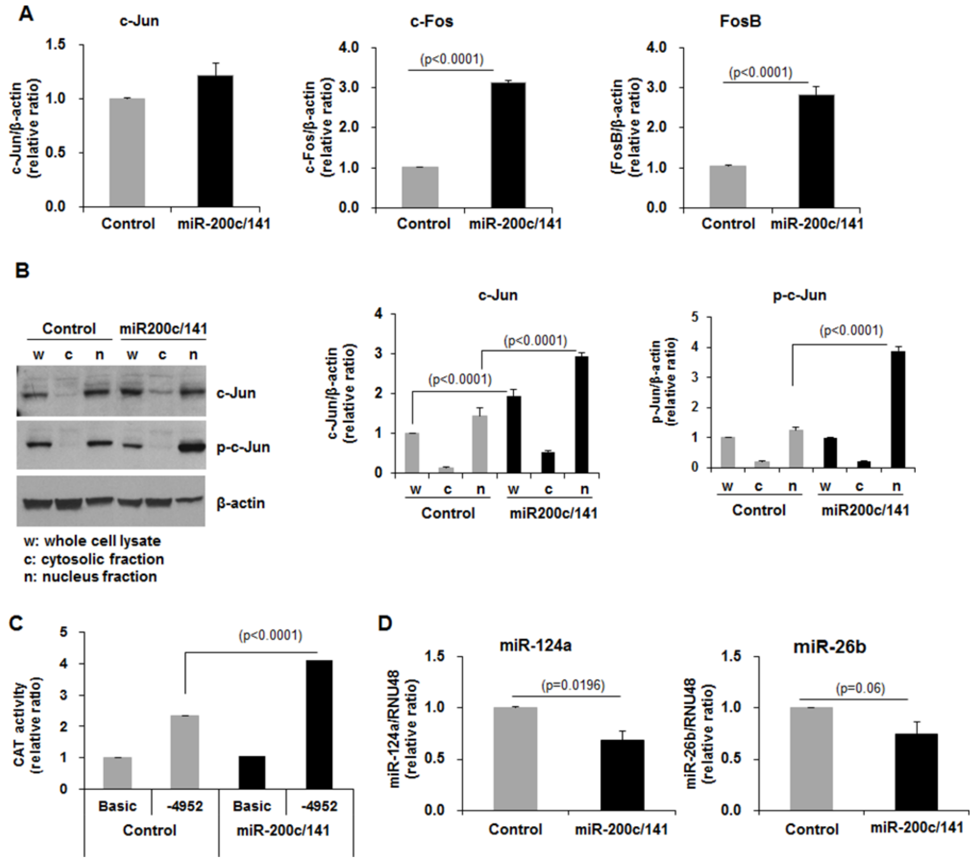

Supplementary Figure 2: miR-200c/141 indirectly increased SerpinB2 by regulating SerpinB2 transcription factors and miRNAs. Real-time RT-PCR showed higher levels of c-Jun, c-Fos and FosB in MDA-MB-231$^{\text{miR-200c/141}}$ cells compared to controls **(A)** Western blotting showed increased nuclear localization of p-c-Jun in MDA-MB-231$^{\text{miR-200c/141}}$ cells compared to controls **(B)** CAT reporter assay revealed increased SerpinB2 promoter activity in MDA-MB-231$^{\text{miR-200c/141}}$ cells compared to controls **(C)** miR-124a and miR-26b were downregulated in MDA-MB-231$^{\text{miR-200c/141}}$ cells compared to controls **(D)** **P*<0.05.

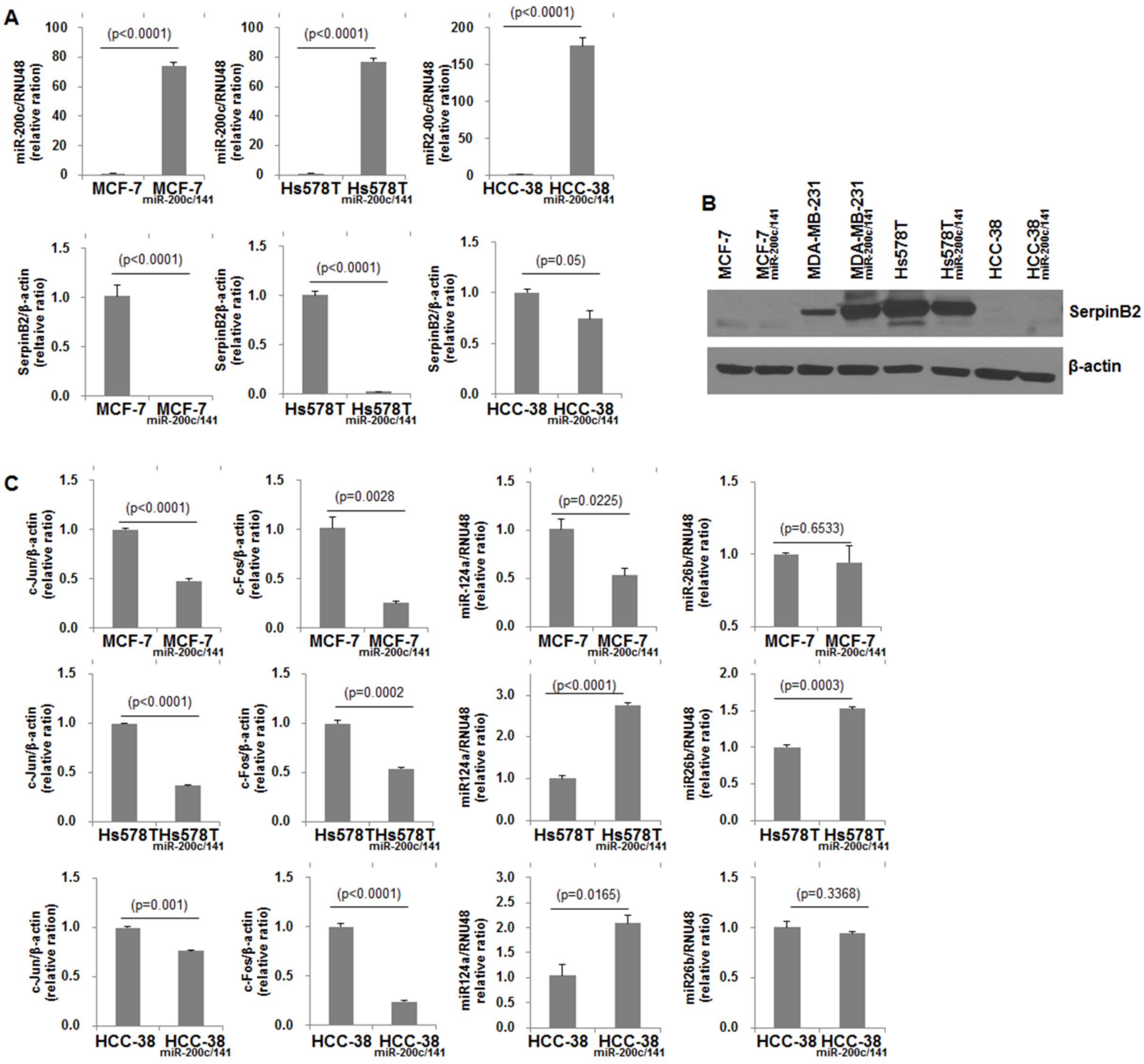

Supplementary Figure 3: Real-time RT-PCR analysis of SerpinB2, c-Jun, c-Fos, miR-200c, miR124a and miR-26b and western blot of SerpinB2 in MCF-7$^{miR\text{-}200c/141}$, Hs578T$^{miR\text{-}200c/141}$, and HCC38$^{miR\text{-}200c/141}$ cells. (**A**) A significant increase in miR-200c levels was observed in MCF-7$^{miR\text{-}200c/141}$, Hs578T$^{miR\text{-}200c/141}$, and HCC-38$^{miR\text{-}200c/141}$ cells compared to controls. SerpinB2 mRNA was downregulated in MCF-7$^{miR\text{-}200c/141}$, Hs578T$^{miR\text{-}200c/141}$, and HCC-38$^{miR\text{-}200c/141}$ cells. (**B**) SerpinB2 protein levels increased in only MDA-MB-231$^{miR\text{-}200c/141}$ cells relative to control. (**C**) c-Jun and c-Fos mRNA levels significantly decreased in MCF-7$^{miR\text{-}200c/141}$, Hs578T$^{miR\text{-}200c/141}$, and HCC-38$^{miR\text{-}200c/141}$ cells. miR-124a significantly decreased in only MCF-7$^{miR\text{-}200c/141}$ cells but increased in Hs578T$^{miR\text{-}200c/141}$ and HCC-38$^{miR\text{-}200c/141}$ cells compared to controls.

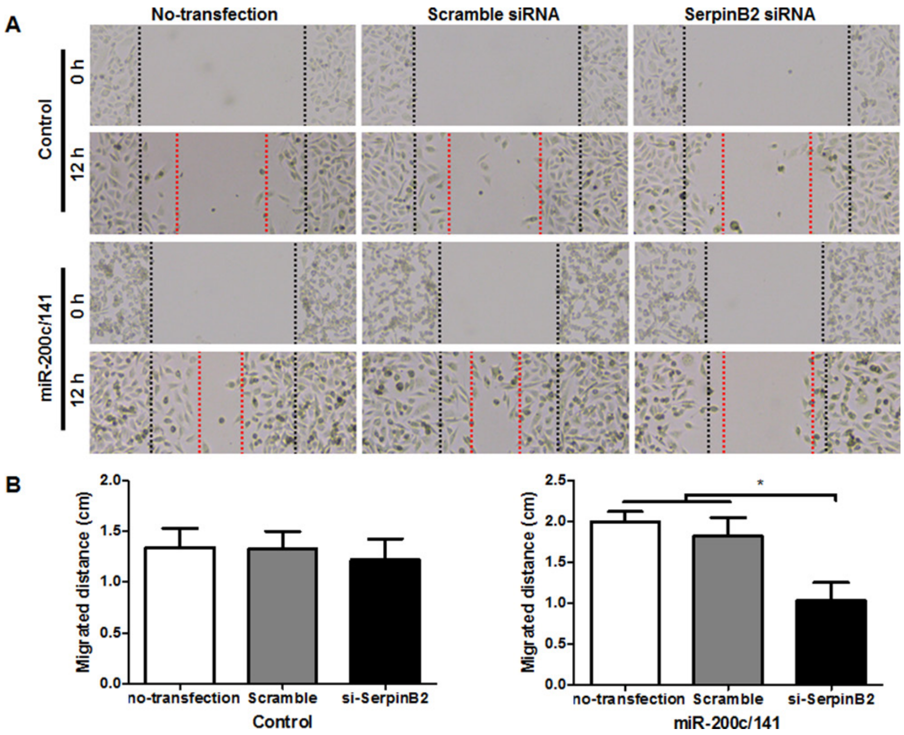

Supplementary Figure 4: SerpinB2 knockdown suppressed MDA-MB-231 cell migration promoted by miR200c/141 cluster overexpression. Representative wound-healing assay images of control and MDA-MB-231$^{miR\text{-}200c/141}$ cells transfected with SerpinB2 or scramble siRNA **(A)** Lateral migration of control (n=3), control+scramble (n=3), control+si-SerpinB2 (n=3), MDA-MB-231$^{miR\text{-}200c/141}$ (n=3), MDA-MB-231$^{miR\text{-}200c/141}$+scramble (n=3), and MDA-MB-231$^{miR\text{-}200c/141}$+si-SerpinB2 cells (n=3) **(B)** *P<0.05.

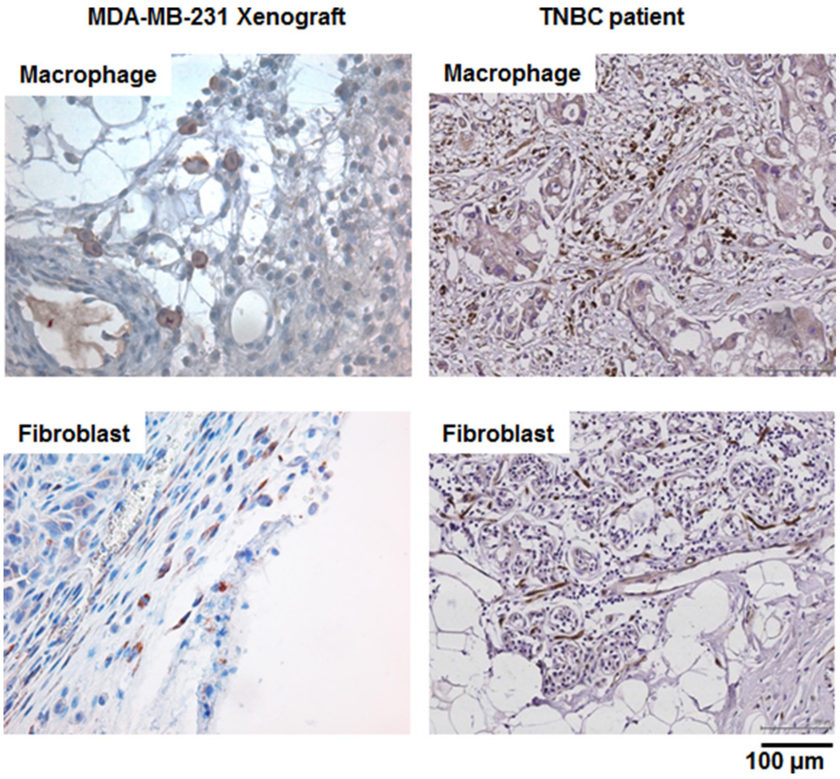

Supplementary Figure 5: Macrophages and fibroblasts associated with MDA-MB-231 xenografts and primary TNBC patient tumors overexpressed SerpinB2. Macrophage and fibroblast populations overexpressing SerpinB2 were observed at or near the tumor border in xenografts and TNBC patient samples.

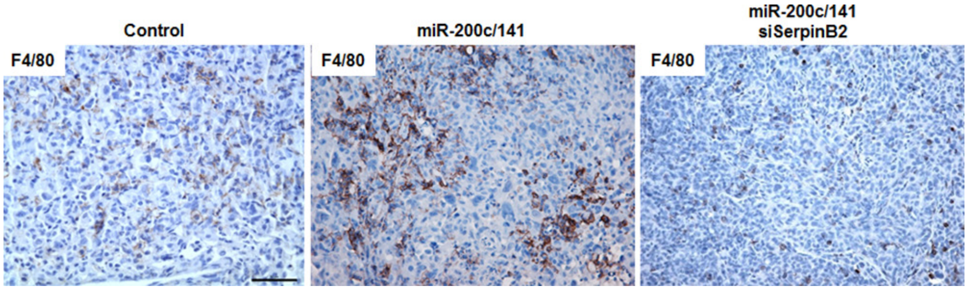

Supplementary Figure 6: SerpinB2 knockdown suppressed macrophages infiltration into MDA-MB-231$^{miR\text{-}200c/141}$ cell xenografts. Macrophage infiltration (F4/80) was observed in MDA-MB-231$^{miR\text{-}200c/141}$ cell tumors, but decreased in SerpinB2 knockdown tumors.

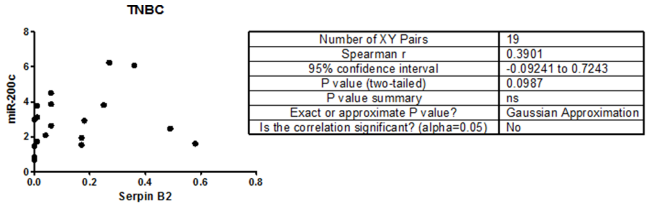

Number of XY Pairs	19
Spearman r	0.3901
95% confidence interval	-0.09241 to 0.7243
P value (two-tailed)	0.0987
P value summary	ns
Exact or approximate P value?	Gaussian Approximation
Is the correlation significant? (alpha=0.05)	No

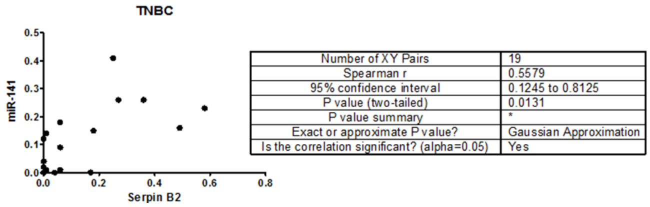

Number of XY Pairs	19
Spearman r	0.5579
95% confidence interval	0.1245 to 0.8125
P value (two-tailed)	0.0131
P value summary	*
Exact or approximate P value?	Gaussian Approximation
Is the correlation significant? (alpha=0.05)	Yes

Supplementary Figure 7: Correlation between miR-200c, miR-141 and SerpinB2 mRNA in primary TNBC tumor tissues. SerpinB2 expression was positively correlated with miR-200c and miR-141 expression in TNBC patients (n=19).

Supplementary Table 1: Real-time RT-PCR primer sequences

Gene	Accession no.		Sequence (5’->3’)
SerpinB2	NM_001143818	Forward<br>Reverse	CCTGATGCGATTTTGCAGGCGCT<br>CGCAGACTTCTCACCAA
SerpinE1	NM_000602	Forward<br>Reverse	CCCCACTTCTTCAGGCTGTT<br>GCCGTTGAAGTAGAGGGCAT
uPA	NM_001145031<br>NM_002658.3	Forward<br>Reverse	TCCAAGAGTGCATGGTGCAT<br>CCTCCACACACGTAGGTGAC
MAL2	NM_052886NM	Forward<br>Reverse	CTGGAGATTCTGTTCGGGGG<br>ATTTGAGCCACCATGCCAGA
C15orf54	NM_207445	Forward<br>Reverse	AGCCACTCATCACATGACGG<br>AGTGCACACACGCTTTGTTG
PLCβ4	NM_001172646	Forward<br>Reverse	GCGGGTACCTTCTCAAACCA<br>GTCAGTGGGCAACCCATACA
MPZL2	NM_144765	Forward<br>Reverse	GAGGAAAGGCTCAACCAAGAGA<br>TGGTTGGAAAACGGGTCACA
LCP1	NM_002298	Forward<br>Reverse	CCTGGCTGATGATTTGTCATTCT<br>ACCAGGAACCCCTTCTTTCTG
KRTAP2-4	NM_033184	Forward<br>Reverse	ATGCCCCCACAGAGCAATAC<br>GTGGGTGAGGGTGGTAATGG
EDN1	NM_001955	Forward<br>Reverse	CACAAAGGCAACAGACCGTG<br>GGTCTCCGACCTGGTTTGTC
ID2	NM_002166	Forward<br>Reverse	ATGAAAGCCTTCAGTCCCGT<br>GAGCTTGGAGTAGCAGTCGT
EGR1	NM_001964	Forward<br>Reverse	CCAACAGTGGCAACACCTTG<br>AAATGTCAGTGTTCGGCGTG
c-Jun	NM_002228.3	Forward<br>Reverse	GAGCTGGAGCGCCTGATAAT<br>CCCTCCTGCTCATCTGTCAC
FosB	NM_005252.3	Forward<br>Reverse	GCGCCGGGAACGAAATAAAC<br>ACCAGCACAAACTCCAGACG
c-Fos	NM_006732.2	Forward<br>Reverse	CGTGCCAGACATGGACCTAT<br>CGGGGTAGGTGAAGACGAAG

Supplementary Table 2: Relationships between miR-200c/141 expression and clinicopathological features

Parameters	No. of cases	miR200c High (%)	miR200c Low (%)	*P*-value	miR141 High (%)	miR141 Low (%)	*P*-value
TNBC	21	10	11		10(47.6)	11(52.4)	
Age				0.80			0.04
≧50	9	4(44.4)	5(55.6)		2(22.2)	7(77.8)	
<50	12	6(50.0)	6(50.0)		8(66.7)	4(33.3)	
Tumor size				0.67			0.46
≧2 cm	3	1(33.3)	2(66.7)		2(66.7)	1(33.3)	
<2 cm	18	4(22.2)	14(77.8)		8(44.4)	10(55.6)	
LN metastasis				0.27			0.83
Negative	11	4(36.3)	7(63.7)		5(45.4)	6(54.6)	
Positive	10	6(60.0)	4(40.0)		5(50.0)	5(50.0)	
TNM stage				0.89			0.18
I-II	15	7(46.7)	8(53.3)		6(40.0)	9(60.0)	
III-IV	6	3(50.0)	3(50.0)		4(60.0)	2(40.0)	
